# The emerging roles and mechanism of N6-methyladenosine (m^6^A) modifications in urologic tumours progression

**DOI:** 10.3389/fphar.2023.1192495

**Published:** 2023-05-22

**Authors:** Wenhao Zhu, Renshan Zhao, Xiaomin Guan, Xu Wang

**Affiliations:** Cancer Center, The First Hospital of Jilin University, Changchun, China

**Keywords:** urologic tumours, N6-methyladenosine, epitranscriptome, posttranscriptional modification, coding RNAs, non-coding RNAs

## Abstract

Prostate cancer (PCa), bladder cancer (BC), and renal cell cancer (RCC) are the most common urologic tumours in males. N6-methyladenosine (m^6^A), adenosine N6 methylation, is the most prevalent RNA modification in mammals. Increasing evidence suggests that m^6^A plays a crucial role in cancer development. In this review, we comprehensively analyzed the influence of m^6^A methylation on Prostate cancer, bladder cancer, and renal cell cancer and the relationship between the expression of relevant regulatory factors and their development and occurrence, which provides new insights and approaches for the early clinical diagnosis and targeted therapy of urologic malignancies.

## 1 Introduction

Urological tumours, primarily including PCa, BC and RCC, are a global threat to human health. Incidence and mortality rates are increasing globally (Sung et al., 2021; Siegel et al., 2022). However, most urological tumour patients are diagnosed at advanced stages owing to the absence of distinct symptoms and signs. Although the risk factors for urological tumours are well-defined, however, the underlying molecular mechanisms remain unclear. Therefore, it is vital to understand urological tumour pathogenesis. These might provide novel targets for the early diagnosis and clinical treatment of urological tumours, which may present an objective basis for clinical decision-making (Wang et al., 2020a; Su et al., 2020; Yankova et al., 2021).

Among more than 100 chemical modifications, m^6^A has been identified as one of the most prevalent chemical forms in eukaryotic RNA (Fu et al., 2014; Lewis et al., 2017). It plays a significant role in various biological functions, including RNA translation, stability (degradation and stabilization), splicing, and nucleus export. This reversible m^6^A RNA modification is coordinated by three groups of proteins: methyltransferases (“writers”), demethylases (“erasers”), and m^6^A-binding proteins (“readers”) (Gilbert et al., 2016; Deng et al., 2018; Dierks et al., 2021). Recently, various studies demonstrated that aberrantly expressed m^6^A modifications are associated with numerous human tumours progression, including tumourigenesis, proliferation, and drug resistance (He et al., 2019; Huang et al., 2020a).

In brief, studies on the interaction between m^6^A and tumours havegradually entered the public view. Numerous studies have established a link between m^6^A variables and the development of urological cancers. However, understanding the association between m^6^A modifications and urological cancers remains unclear. Therefore, a systematic review of the molecular mechanisms underlying m^6^A regulation in PCa, BC, and RCC progression is strongly recommended.

This review outlines recent findings on m^6^A modification, relationships, biological activities, and mechanisms of its related proteins in the malignant progression of these three urologic tumours.

## 2 m^6^A modification

**FIGURE 1:**
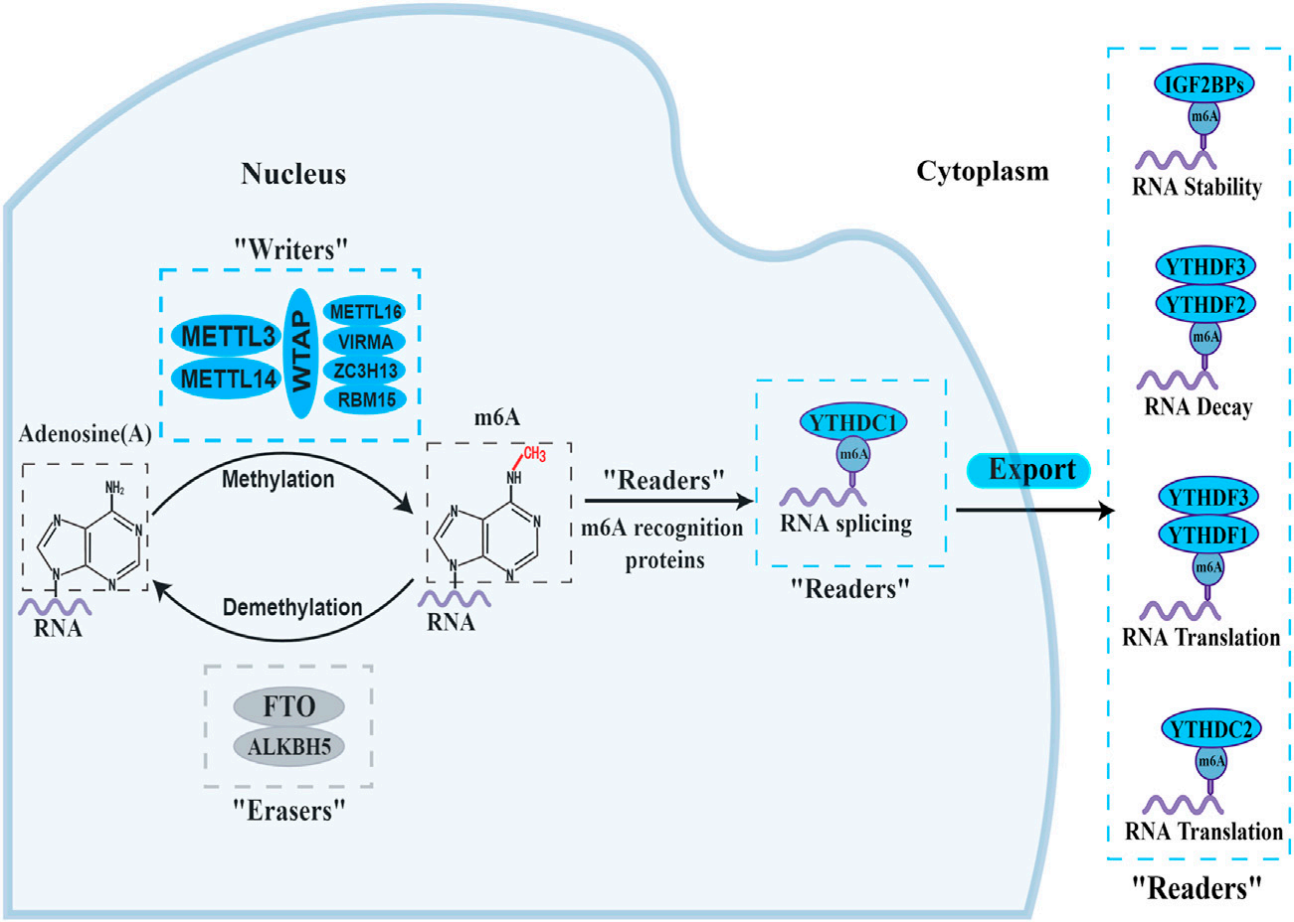
The biology functions of m6A regulators. The three kinds of m6A enzymes are as follows: “writers” (METTL3, METTL14, WTAP, METTL16, VIRMA, ZC3H13 and RBM15), “erasers” (FTO and ALKBH5), and “readers” (YTHDF1/2/3, YTHDC1/2 and IGF2BP1/2/3). They regulate the deposition of m6A modification and affect the translation, stability (degradation and stabilization) and splicing of RNA.

In the process of m^6^A modification, it, mainly occurs in adenines in the consensus motif RRACH(H = A,C or U,R = G or A) with enrichment more prone to be detected in the 3′-untranslated regions (Bodi et al., 2010; Meyer et al., 2012). The deposition, recognition and removal of m^6^A are carried out respectively by regulators “writers”, “readers” and “erasers”. First, the deposition of m^6^A in mRNA is installed and catalysed by a complex consisting of METTL3, METTL14 and WTAP (“writers”). Next, the m^6^A-binding proteins (“readers”), all of which can selectively recognize and bind to methylated mRNA, controlling gene expression via regulating diverse processes, including mRNA maturation, export, translation, and decay, it depends on different kinds of “readers”. Beyond this, the methylation of m^6^A can be removed passively from the transcriptome through degradation of modified RNA by “readers” or actively through FTO and ALKBH5 demethylases. A diagram of the methylation processes involved in m^6^A and their biological functions are displayed in Figure 1. This presents “writers” methylating RNA at the amino group, which is located at position N6 of the adenine nucleobase. As soon as m^6^A is deposited on RNA, the recruitment of m^6^A recognition proteins influences the fate of the RNA, including as translation effectiveness, stability (degradation and stabilization) and splicing, which ultimately affect gene expression. Additionally, “erasers” demethylate m^6^A to remove it from RNA.

At present, “writers” have been extensively studied most. The m^6^A methylase complex comprises a range of methyltransferases. Methyltransferase-like 3 (METTL3), methyltransferase-like 14 (METTL14), and wilm’s tumour-associated protein (WTAP) are the core components of complex (Bokar et al., 1997; Ping et al., 2014; Schwartz et al., 2014). The METTL3 and METTL14 form a heterodimer (Wang et al., 2017). With the help of WTAP, the METTL3-METTL14 heterodimer catalyzes the covalent transfer of a methyl group to adenine. In addition, several other multicomponent methyltransferase complexes have recently been identified and characterized. Such as Methyltransferase-like 16 (METTL16), vir-like m^6^A methyltransferase-associated protein (VIRMA), zinc finger CCCH domain-containing protein 13 (ZC3H13), and RNA binding pattern protein 15 (RBM15) (Melstrom and Chen, 2020; Oerum et al., 2021).

“Erasers", or m^6^A demethylases, can reverse the dynamic m^6^A methylation in the nucleus, including α-ketoglutarate-dependent dioxygenase homolog 5 (ALKBH5), fat mass and obesity-related protein (FTO) (Zheng et al., 2013; Zhao et al., 2014). They all belong to the α-ketoglutarate-dependent dioxygenase family. The discovery of m^6^A demethylases has confirmed that m^6^A modification is dynamically reversible.

“Readers”, or m^6^A recognition proteins, could recognize and bind to m^6^A modification sites and participate in diverse stages of RNA metabolism. Different readers have different biological functions. The most well-known m^6^A readers are the insulin-like growth factor 2 mRNA binding protein (IGF2BP) and the YT521-B homology (YTH) domain families. The IGF2BP family includes IGF2BP1, IGF2BP2, and IGF2BP3, which can improve the stability and translation of the target RNAs (Huang et al., 2018). YTHDF1-3 and YTHDC1-2 were the five main YTH domain proteins. YTHDC1, which is localized in the nucleus, regulates splicing and nuclear export, whereas YTHDC2 enhances the translation efficiency of target RNA. YTHDF3 coordinates with YTHDF1 to promote the translation of target RNA, whereas YTHDF3 cooperates with YTHDF2 to induce target RNA degradation (Shi et al., 2017; Einstein et al., 2021). These proteins create an effective m^6^A regulatory network and play a crucial role in m^6^A modification.

## 3 Role of m^6^A regulators in the progression of PCa

**FIGURE 2:**
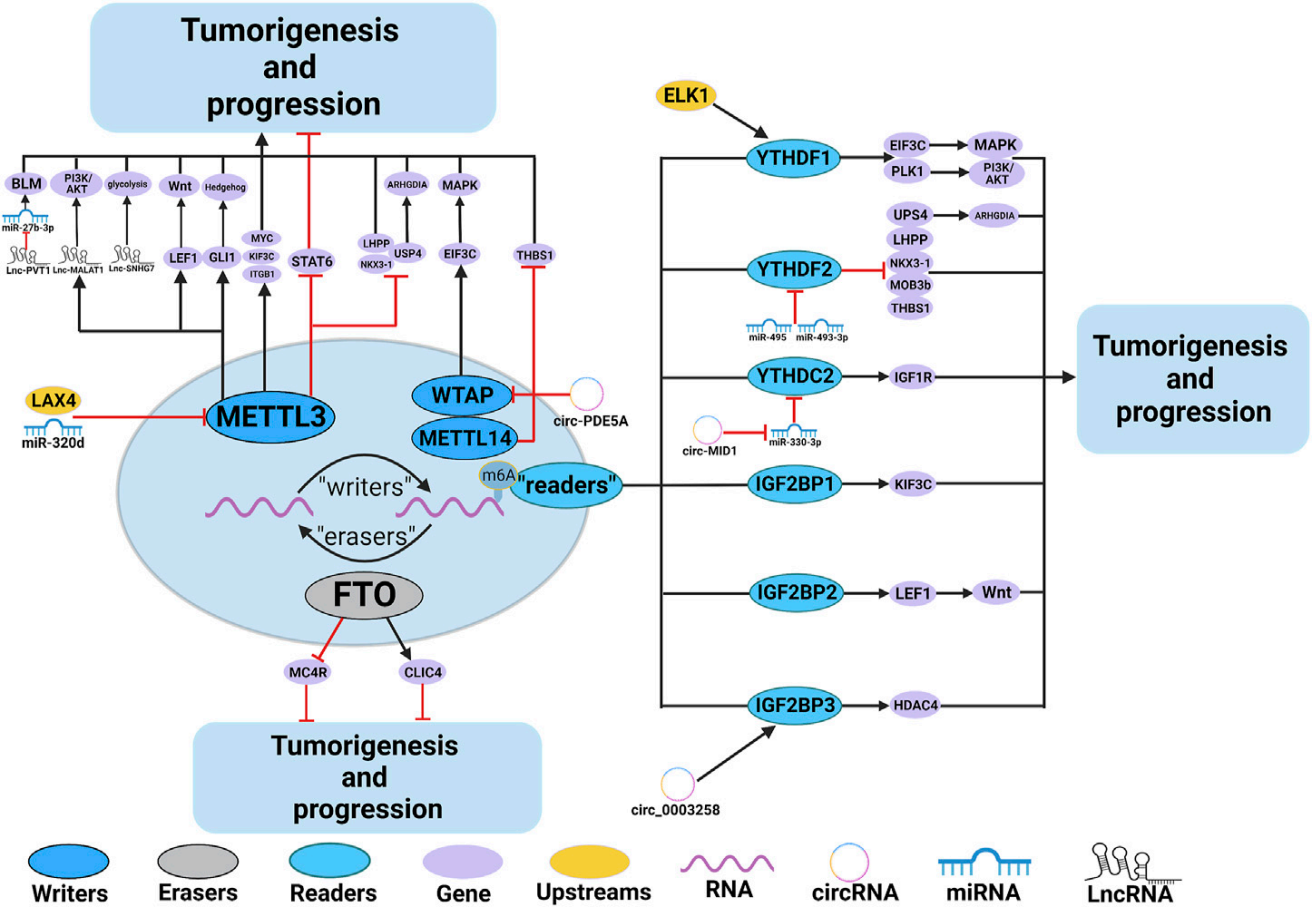
In PCa, m^6^A regulatory proteins contribute to tumorigenesis and progression by interacting with various RNAs. METTL3 and WTAP stimulate the progression of PCa by promoting the expression levels of Lnc-PVT1, Lnc-MALAT1, Lnc-SNHG7, MYC, LEF1, GLI1, ITGB, KIFEC and EIF3C. In addition, miR-320d and circ-PDE5A inhibit the expression of METTL3 and WTAP, leading to PCa suppression. METTL3 and METTL14 stimulate the progression of PCa by inhibiting the expression levels of USP4, NKX3-1, LHPP and THBS1. METTL3 suppresses the progression of PCa by inhibiting the expression levels of STAT6. LXA4 inhibits the expression of METTL3, leading to PCa progression. FTO suppress the progression of PCa by affecting the expression levels of MC4R and CLIC4. YTHDF1, YTHDC2, and IGF2BPs stimulate the progression of PCa by promoting the expression of EIF3C, PLK1, IGF1R, KIF3C, LEF1 and HDAC4. YTHDF2 stimulate the progression of PCa by inhibiting the expression of USP4, LHPP, NKX3-1, MOB3b and THBS1. In addition, ELK1 and circ_0003258 promote the expression of YTHDF1 and IGF2BP3, leading to PCa progression. circ-MID1 could sponge miR-330–3p to promote YTHDC2 expression.

Numerous studies have demonstrated the involvement of m^6^A regulators in various functions of PCa, such as the metabolism of non-coding RNAs (ncRNAs) and M2 macrophage polarization. The roles and mechanisms of m^6^A regulators in PCa are summarized in Figure 2 and Table 1.

**TABLE 1:** The role and mechanism of m6A regulators in PCa.

m6A Regulators	Roles in m6A	Expression in cancer	Mechanism	Function in cancer	References
METTL3	Writer	-	METTL3/Lnc-PVT1/miR-27b-3p/BLM	Promotes tumour tumorigenesis and progression	Chen et al. (2023)
METTL3	Writer	-	miR-320d/METTL3/KIF3C/IFG2BP1	Promotes tumour tumorigenesis and progression	Ma et al. (2021)
METTL3	Writer	upregulation	METTL3/Lnc-MALAT1/PI3K/AKT	Promotes tumour tumorigenesis and progression	Mao et al. (2022)
METTL3	Writer	upregulation	METTL3/Lnc-SNHG7/SRSF1/c-Myc/glycolysis	Promotes tumour tumorigenesis, progression and glycolysis	Liu et al. (2022a)
METTL3	Writer	upregulation	METTL3/LHPP/NKX3-1/YTHDF2/AKT	Promotes tumour tumorigenesis and progression	Li et al. (2020a)
METTL3	Writer	upregulation	METTL3/USP4/YTHDF2/ELAV1/ARGFDIA	Promotes tumour tumorigenesis and progression	Chen et al. (2021a)
METTL3	Writer	upregulation	METTL3/MYC	Promotes tumour tumorigenesis and progression	Yuan et al. (2020)
METTL3	Writer	upregulation	METTL3/LEF1/IGF2BP2/Wnt	Promotes tumour tumorigenesis and progression	Ma et al. (2020)
METTL3	Writer	upregulation	METTL3/GLI1/Hedgehog	Promotes tumour tumorigenesis and progression	Cai et al. (2019)
METTL3	Writer	upregulation	METTL3/ITGB1	Promotes tumour tumorigenesis, progression and bone metastasis	Li et al. (2020b)
METTL3	Writer	downregulation	LAX4/METTL3/STAT6/M2macrophage polarization	Inhibits tumour progression and M2macrophage polarization	Jia et al. (2022)
METTL14	Writer	upregulation	METTL14/THBS1/YHTDF2	Promotes tumour tumorigenesis and progression	Wang et al. (2022b)
WTAP	Writer	-	circ-PDE5A/WTAP/YTHDF1/EIF3C/MAPK	Promotes tumour tumorigenesis and progression	Ding et al. (2022a)
FTO	Eraser	downregulation	FTO/MC4R	Inhibits tumour progression and migration	Li and Cao (2022)
FTO	Eraser	downregulation	FTO/CLIC4	Inhibits tumour progression and migration	Zou et al. (2022)
YTHDF1	Reader	-	circ-PDE5A/WTAP/YTHDF1/EIF3C/MAPK	Promotes tumour tumorigenesis and progression	Ding et al. (2022a)
YTHDF1	Reader	upregulation	ELK1/YTHDF1/PLK1/PI3K/AKT	Promotes tumour tumorigenesis and progression	Li et al. (2022a)
YTHDF2	Reader	upregulation	METTL3/LHPP/NKX3-1/YTHDF2/AKT	Promotes tumour tumorigenesis and progression	Li et al. (2020a)
YTHDF2	Reader	upregulation	METTL3/USP4/YTHDF2/ELAV1/ARGFDIA	Promotes tumour tumorigenesis and progression	Chen et al. (2021a)
YTHDF2	Reader	-	METTL14/THBS1/YHTDF2	Promotes tumour tumorigenesis and progression	Wang et al. (2022b)
YTHDF2	Reader	upregulation	miR-493–3p/YTHDF2	Promotes tumour tumorigenesis and progression	Li et al. (2018)
YTHDF2	Reader	upregulation	KDM5a/miR-495/YTHDF2/MOB3b	Promotes tumour tumorigenesis and progression	Du et al. (2020a)
YTHDC2	Reader	upregulation	circ-MID1/miR-330–3p/YTHDC2/IGF1R/AKT	Promotes tumour tumorigenesis and progression	Ding et al. (2022b)
IGF2BP1	Reader	-	miR-320d/METTL3/KIF3C/IFG2BP1	Promotes tumour tumorigenesis and progression	Ma et al. (2021)
IGF2BP2	Reader	upregulation	METTL3/LEF1/IGF2BP2/Wnt	Promotes tumour tumorigenesis and progression	Ma et al. (2020)
IGF2BP3	Reader	upregulation	Circ-0003258/HDAC4/IGF2BP3/ERK	Promotes tumour metastasis	Yu et al. (2022)

### 3.1 m^6^A writers and PCa

The m^6^A regulatory factors are essential for PCa tumourigenesis and progression. Studies of writers on PCa have primarily focused on the regulatory component METTL3. Recent studies have indicated that METTL3 plays an oncogenic role in PCa (Cai et al., 2019; Li et al., 2020a; Li et al., 2020b; Ma et al., 2020; Yuan et al., 2020; Chen et al., 2021a; Ma et al., 2021; Liu et al., 2022a; Wang et al., 2022a; Mao et al., 2022; Chen et al., 2023). Only one study has suggested that it is a carcinogenic tumour suppressor (Jia et al., 2022).

Recent studies have shown that METTL3 can affect the synthesis and function of various ncRNAs, such as miRNAs and LncRNAs. Moreover, ncRNAs also have an important regulatory effect on METTL3 in PCa progression (Zeng et al., 2020). Chen et al. (Chen et al., 2023) reported that METTL3 could upregulate the Lnc-plasmacytoma variant translocation 1 (PVT1) expression in an m^6^A-dependent manner. Further investigation showed that PVT1 serve as a ceRNA to isolate miR-27b-3p and promote PCa progression via the METTL3/PVT1/miR-27b-3p/Bloom syndrome protein (BLM) axis. In addition, a study indicated that Kinesin Family Member 3C (KIF3C) was overexpressed in PCa and negatively correlated with patient outcomes (Ma et al., 2021). The m^6^A writer METTL3 can promote m^6^A modification of KIF3C, thus enhancing KIF3C mRNA stabilization and expression by IGF2BP1. miR-320d, as a tumour-suppressor factor and upstream target of METTL3, inhibits KIF3C expression by targeting METTL3 and curbing PCa progression. As a lncRNA, the metastasis-associated lung adenocarcinoma transcript 1 (MALAT1) is oncogenic in numerous tumours progressions (Fan et al., 2014; Jen et al., 2017). Yuan et al. (Mao et al., 2022) confirmed that METTL3 could upregulate the m^6^A level of LncRNA-MALAT1, which activates the PI3K/AKT signaling pathway and promotes the growth and invasion of PCa. The molecular mechanisms of m^6^A methylation modifications in tumour cells have recently been studied. Liu et al. (Liu et al., 2022a) reported upregulation of lncRNA small nucleolar RNA host gene 7 (SNHG7) induced by m^6^A. METTL3 promoted the m^6^A modification of SNHG7 and enhanced its stability. Upregulated SNHG7 expression could accelerate glycolysis via the serine/arginine-rich splicing factor 1 (SRSF1)/c-Myc axis and promote the progression of PCa.

Recent studies revealed that m^6^A modification could affect tumour formation by regulating the m^6^A modification in the mRNAs of oncogenes, tumour suppressors, and transcription factors (An and Duan, 2022). Li et al.demonstrated that two tumour suppressors, the phospholysine phosphohistidine inorganic pyrophosphate phosphatase (LHPP) and NK3 Homeobox 1 (NKX3-1), are downregulated in PCa (Li et al., 2020a). Further investigation verified that YTHDF2 could recognize the abundance of m^6^A-modified mRNA of LHPP and NKX3-1 induced by METTL3 and degrade them, thereby promoting AKT phosphorylation and tumour progression. Chen et al. (Chen et al., 2021a) reported that YTHDF2 could recognize and degrade the abundance of m^6^A-modified Ubiquitin Specific peptidase 4 (USP4) mRNA promoted by METTL3 in the PCa cells. The decreased USP4 mRNA could enhance Rho GDP dissociation inhibitor α (ARHGDIA) expression via the METTL3-USP4-ELAVL1 axis, which finally promotes the progression of PCa cells. In addition, researchers elucidated that MYC(c-myc) was a target of METTL3-mediated m^6^A modification and its expression was enhanced by METTL3 in PCa (Yuan et al., 2020). This leads to PCa’s oncogenic functions. Several signaling pathways and molecules involved in tumour development have been identified as m^6^A targets and controlled by m^6^A modifiers. According to Ma et al. (Ma et al., 2020), a significant upregulation of Lymphoid Enhancer-binding Factor 1 (LEF1) expression was induced by increased METTL3 in PCa patients. Increased METTL3 and LEF1 levels activate the Wnt pathway and promote tumour progression. Similarly, the increased expression of METTL3 in PCa could enhance the expression of Glioma-associated Oncogene Homolog 1 (GLI1) via an m^6^A catalytic activity-dependent manner (Cai et al., 2019). The GLI1 overexpression activates Hedgehog Pathway and promotes tumour growth.

Tumour microenvironments (TMEs) are a complex microenvironment of cancer, stromal, and immune cells (Binnewies et al., 2018). Recently, much research has been conducted to study the tumour microenvironment recently. However, little is known about the role of m^6^A in regulating the immune microenvironment of tumours. Macrophages are the most prevalent immune cells in the microenvironment of tumour (Condeelis and Pollard, 2006). Studies have revealed that the total m^6^A levels were considerably downregulated in macrophages induced by the downregulation of METTL3 in PCa. According to Jia et al. (Jia et al., 2022), prostate cancer cell-derived small lipid molecule lipoxin A4 (LXA4) could inhibit METTL3, and the decreased METTL3 promotes signal transducers and activators of transcription 6 (STAT6) phosphorylation, which in turn induces M2 macrophage polarization and enhances tumour progression. This indicates that METTL3 could also act as a tumour suppressor in PCa. m^6^A modification also accounts for TME remodeling in tumours, including remodeling in organs with tumour metastasis. Li et al. (Li et al., 2020b) reported that elevated METTL3 levels in PCa upregulated and enhanced the integrin β1 (ITGB1) mRNA’s stability and expression in an m^6^A-HuR-dependent manner. The overexpressed ITGB1 promotes bone metastasis by improving binding to Collagen I and tumour cell motility in PCa.

Wang et al. (Wang et al., 2022b) revealed that METTL14 is upregulated and oncogenic in PCa. Thrombospondin 1 (THBS1), a tumour suppressor, was identified as the target gene of METTL14. YTHDF2 recognizes methylated THBS1 mRNA induced by METTL14 and inhibits its expression. Circ-PDE5A is found downregulated and acts as a metastasis suppressor in PCa. Ding et al. (Ding et al., 2022a) reported that circ-PDE5A inhibited the Eukaryotic Initiation Factor 3 (EIF3C) expression by binding to WTAP and inhibiting its m^6^A methylation ability. Moreover, the decreased m^6^A levels of EIF3C mRNA also inhibited the recognition by YTHDF1, which curbed the MAPK pathway. WATP plays an oncogenic role in PCa.

### 3.2 m^6^A erasers and PCa

Studies on m^6^A erasers in PCa have focused on FTO. As an m^6^A demethylase, FTO is downregulated in PCa, and its level is negatively correlated with advanced tumour stage (Zhu et al., 2021; Li and Cao, 2022; Zou et al., 2022). Cao et al. (Li and Cao, 2022) reported that FTO downregulates the melanocortin-4 receptor (MC4R) in an m^6^A-dependent manner to inhibit PCa tumour progression and migration. The reduction of chloride intracellular channel 4 (CLIC4) in cancer cells is common in human tumours and signals the progression and invasion of malignant cells (Suh et al., 2007). However, the mechanism by which CLIC4 expression in PCa is regulated by m^6^A remains unclear. According to a recent study (Zou et al., 2022), FTO suppressed PCa proliferation and metastasis by reducing the degradation of CLIC4 mRNA in an m^6^A-dependent manner, which provided new insights into the mechanism of PCa.

### 3.3 m^6^A readers and PCa

In PCa, studies on the m^6^A readers mainly focused on YTHDF1, YTHDF2, YTHDC2, and IGF2BPs. Recent studies have reported that YTHDF1 is upregulated in PCa tissues compared to those normal tissues and is correlated with poor outcomes (Li et al., 2021; Li et al., 2022a).

Polo-like kinase 1 (PLK1) regulates mitotic processes, leading to cellular proliferation. There is a substantial relationship between PLK1 overexpression and certain human cancers (Lee et al., 2015). However, the relationship between m^6^A regulation and PLK1 in PCa remains unclear. Li et al. (Li et al., 2022a) reported that the transcription factor ELK1 was overexpressed in PCa tissues and enhanced the YTHDF1 transcription. Further investigation showed that PLK1 is an m^6^A modification downstream target of YTHDF1 and is facilitated by it. This activates the PI3K/AKT signaling and drives the progression of PCa.

Some researchers have identified the tumour-promoting effect of YTHDF2 by investigating its upstream signaling in PCa. Some of these studies have been described in the previous sections (Li et al., 2020a; Wang et al., 2022b). Several studies have shown that miRNA processing and maturation are significantly affected by m^6^A modifications (Chen et al., 2020a). In lieu, miRNAs can also affect m^6^A synthesis. miR-493–3p and miR-495, tumour suppressor miRNAs in PCa, have been reported to regulate the expression of the YTHDF2. YTHDF2 is a direct downstream target of miRNAs (Li et al., 2018; Du et al., 2020a). The down-expressed miR-493–3p in PCa could enhance the expression of YTHDF2, thereby promoting the progression of PCa by indirectly regulating m^6^A levels. (Li et al., 2018). Chen et al. (Du et al., 2020a) showed that miR-495 is inhibited and downregulated by lysine demethylase 5a (KDM5a), an H3K4me3 demethylase overexpressed in PCa. Further investigation proved that YTHDF2 was a downstream target of miR-495, and the upregulated YTHDF2 could inhibit the expression of MOB kinase activator 3 B (MOB3B) by recognizing m^6^A modification of MOB3B mRNA and inducing mRNA degradation, which consequently promoted tumour tumourigenesis and progression in PCa through an m^6^A-dependent way. These findings demonstrated the roles of miRNA and YTHDF2 in the m^6^A modification and development of PCa and described how the m^6^A-related regulatory factors in PCa were activated, revealing a deeper understanding of the mechanism of PCa.

Recent studies have confirmed that some circRNAs play a crucial role in the progression of PCa (Xiang et al., 2019; Kong et al., 2020), and several studies have assessed the potential molecular mechanism of circRNAs with m^6^A modifications in PCa. Type 1 insulin-like growth factor receptor (IGF1R), an oncogene, is considered to be involved in the tumour progression by mediating the ATK signaling pathway; however, the specific mechanism of IGF1R in PCa is unclear (Li et al., 2014; Du et al., 2020b). Ding et al. (Ding et al., 2022b) reported that circRNA midline-1 (circ-MID1) was overexpressed and functioned to sponge miR-330–3p in PCa cells. MiR-330–3p plays an anti-tumour role in many cancers (Jin et al., 2019a; Huang et al., 2020b), including PCa (Li et al., 2020c). Further investigations demonstrated that YTHDC2 and IGF1R are the downstream targets of miR-330–3p. Therefore, circ-MID1 may promote the progression of PCa by regulating the miR-330–3p/YTHDC2/IGF1R/AKT axis. Additionally, Yu et al. (Yu et al., 2022) reported that overexpressed circ-0003258 in PCa could bind to IGF2BP3, increase HDAC4 mRNA stability, and activate the ERK signaling pathway, thereby accelerating PCa metastasis.

## 4 Role of m^6^A regulators in the progression of BC

**FIGURE 3:**
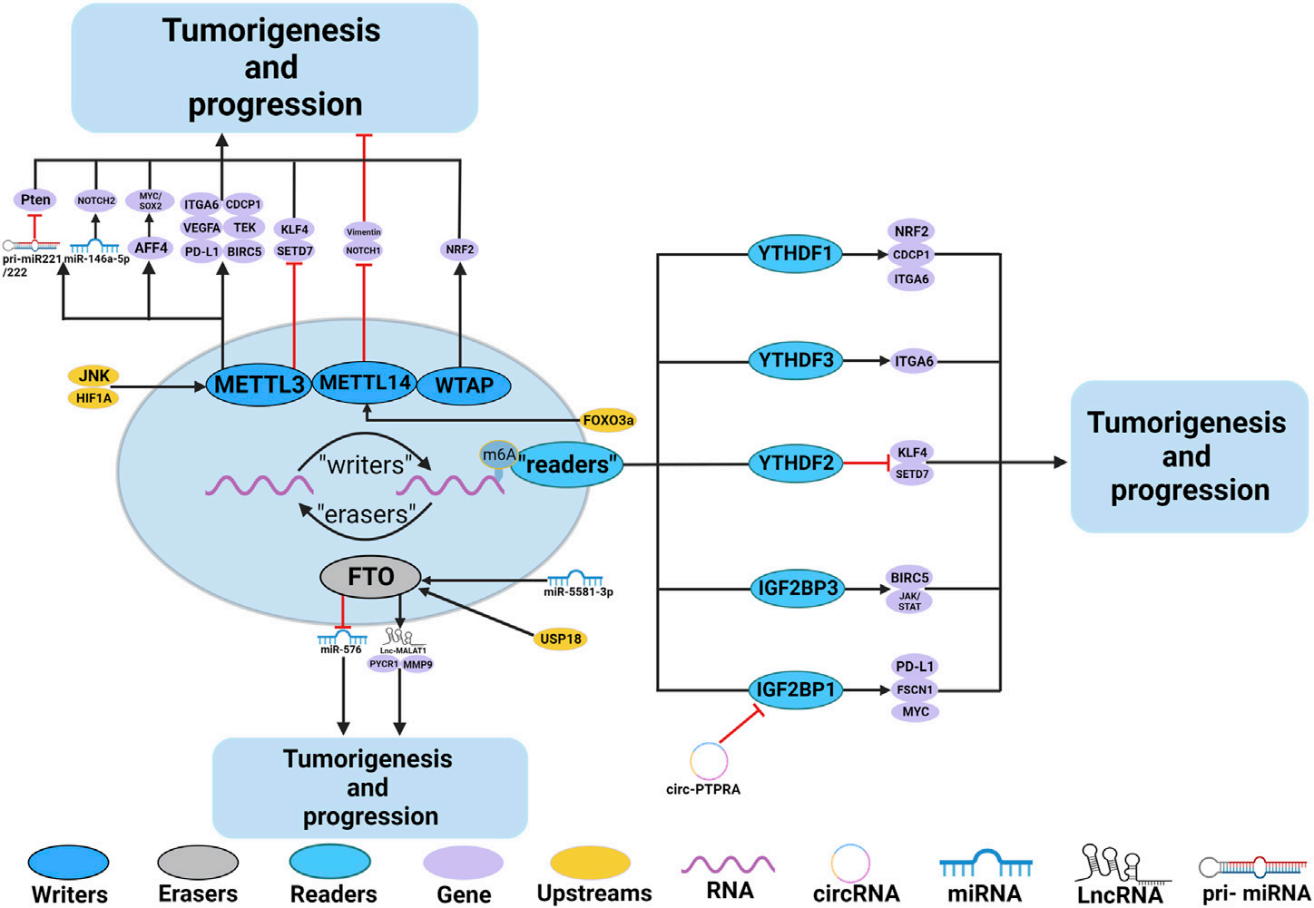
In BC, m^6^A regulatory proteins contribute to tumorigenesis and progression by interacting with various RNAs. METTL3 and WTAP stimulate the progression of BC by promoting the expression levels of PD-L1, BIRC5, miR-146a-5p, pri-miR221/222, TEK, VEGF-A, ITGA6, CDCP1, AFF4 and NRF2. Upstream regulators JNK and HIF1A promote the expression of METTL3, leading to BC progression. METTL3 promote the progression of BC by inhibiting the expression levels of KLF4 and SETD7. METTL14 suppresses the progression of BC by inhibiting the expression levels of Vimentin and Notch1. FTO promote the tumorigenesis and progression of BC by regulating the expression of Lnc-MALAT1, miR-576, MMP9 and PYCR1. In addition, miR-5581–3p inhibits the expression of FTO, leading to BC suppression. USP18 promotes the expression of FTO, leading to BC progression. YTHDF1, YTHDF3, IGF2BP1 and IGF2BP3 stimulate the progression of BC by promoting the expression of NRF2, CDCP1, ITGA6, PD-L1, FSCN1, MYC, BIRC5 and JAK/STAT. In addition, circ-PTPRA inhibits the expression of IGF2BP1, leading to BC suppression. YTHDF2 promote the progression of BC by inhibiting the expression levels of KLF4 and SETD7.

Numerous studies have demonstrated the participation of m^6^A regulators in BC, such as immune escape and EMT. The roles and mechanisms of m^6^A regulators in BC are summarized in Figure 3 and Table 2.

**TABLE 2:** The role and mechanism of m6A regulators in BC.

m6A Regulators	Roles in m6A	Expression in cancer	Mechanism	Function in cancer	References
METTL3	Writer	upregulation	JNK/c-jun/METTL3/PD-L1/IGF2BP1	Promotes tumour immune escape	Ni et al. (2022)
METTL3	Writer	upregulation	PM2.5/HIF1A/METTL3/BIRC5/IGF2BP3/VEGFA	Promotes tumour progression, metastasis and angiogenesis	Liu et al. (2022b)
METTL3	Writer	upregulation	METTL3/miR-146a-5p/Numb/NOTCH2	Promotes tumour progression	Yi et al. (2020)
METTL3	Writer	upregulation	METTL3/pri-miR221/222/DGCR8	Promotes tumour proliferation	Han et al. (2019a)
METTL3	Writer	upregulation	METTL3/TEK/VEGF-A	Promotes tumour angiogenesis	Wang et al. (2021)
METTL3	Writer	upregulation	METTL3/ITGA6/YTHDF1/3	Promotes tumour progression and development	Jin et al. (2019b)
METTL3	Writer	upregulation	METTL3/SETD7/KLF4/YTHDF2	Promotes tumour progression	Xie et al. (2020)
METTL3	Writer	upregulation	METTL3/CDCP1/YTHDF1	Promotes tumorigenesis	Khan et al. (2021)
METTL3	Writer	upregulation	METTL3/AFF4/SOX2/MYC	Promotes tumorigenesis	Gao et al. (2020)
METTL14	Writer	downregulation	maintenance the methyltransferase complex and m6A abundance	Promotes tumour progression	Guimaraes-Teixeira et al. (2022)
METTL14	Writer	downregulation	METTL14/Notch1/TICs	Inhibits tumour progression and tumorigenesis	Gu et al. (2019)
METTL14	Writer	downregulation	ISO/FOXO3a/METTL14/Vimentin	Inhibits tumour EMT	Zhang et al. (2021a)
WTAP	Writer	upregulation	WTAP/NRF2/YTHDF1	Inhibits ferroptosis and promotes tumour malignancy	Wang et al. (2023)
FTO	Eraser	downregulation	influence the FTO-mediated m6A RNA demethylation	Inhibits tumour tumorigenesis, proliferation and invasion	Sun et al. (2022)
FTO	Eraser	upregulation	FTO/Lnc-MALAT1/miR-384/MAL2	Promotes the viability and tumorigenesis of tumour cells	Tao et al. (2021)
FTO	Eraser	upregulation	FTO/miR-576/CDK6	Promotes tumour proliferation, migration and invasion	Zhou et al. (2021)
FTO	Eraser	upregulation	miR-5581–3p/FTO/MMP9	Promotes tumour progression and proliferation	Sun et al. (2022)
FTO	Eraser	upregulation	USP18/FTO/PYCR1	Promotes tumorigenesis and progression	Song et al. (2021)
YTHDF1/YTHDF3	Reader	-	METTL3/ITGA6/YTHDF1/3	Promotes tumour progression and development	Hamidi and Ivaska (2018)
YTHDF1	Reader	-	METTL3/CDCP1/YTHDF1	Promotes tumorigenesis	Khan et al. (2021)
YTHDF1	Reader	upregulation	WTAP/NRF2/YTHDF1	Promotes tumour progression	Wang et al. (2023)
YTHDF2	Reader	upregulation	METTL3/SETD7/KLF4/YTHDF2	Inhibits ferroptosis and promotes tumour malignancy	Xie et al. (2020)
IGF2BP1	Reader	-	JNK/c-jun/METTL3/PD-L1/IGF2BP1	Promotes tumour immune escape	Ni et al. (2022)
IGF2BP1	Reader	upregulation	circ-PTPRA/IGF2BP1/FSCN1/MYC	Promotes tumour proliferation, migration and invasion	Xie et al. (2021a)
IGF2BP3	Reader	upregulation	PM2.5/HIF1A/METTL3/BIRC5/IGF2BP3/VEGFA	Promotes tumour progression, metastasis and angiogenesis	Liu et al. (2022b)
IGF2BP3	Reader	upregulation	IGF2BP3/JAK/STAT	Promotes tumour proliferation and tumorigenesis	Huang et al. (2020d)

### 4.1 m^6^A writers and BC

m^6^A regulatory factors are essential for BC tumourigenesis and progression. Studies on writers of BC have mostly focused on the regulatory factor METTL3. The results showed that METTL3 was significantly upregulated in bladder cancer cell lines and was identified as an oncogene. Poor prognosis was also associated with high levels of METTL3 in BC. Knockdown of METTL3 in bladder cancer cells rendered it less proliferative, invasive, and viable (Chen et al., 2019a; Han et al., 2019a).

In recent years, the components of TME have been increasingly recognized as effective tumour immunotherapy targets (Bader et al., 2020; Sadeghi Rad et al., 2021). In addition, m^6^A modification has become increasingly important in anti-tumour immunotherapy (Han et al., 2019b; Wang et al., 2020b). Exploring their association and mechanisms is the premise of individualized precision treatment. Ni et al. (Ni et al., 2022) reported that activated JNK signaling is associated with increased METTL3 expression in BC, and inhibition of JNK signaling reduced the expression of METTL3 and Programmed death ligand 1 (PD-L1). Further studies showed that IGF2BP1 could recognize methylated PD-L1 mRNA induced by overexpressed METTL3 and promote its translation. The JNK/c-jun/METTL3/PD-L1/IGF2BP1 axis contributes to tumour immune escape in an m^6^A-dependent manner in BC.

Long-term exposure to fine particulate matter (PM2.5) is associated with multiple malignancies, including BC. However, the specific mechanisms of oncogenic processes in BC remain unclear. Liu et al. (Liu et al., 2022b) discovered a new mechanism underlying PM2.5 involvement in BC by m^6^A modification. They found that by hypomethylating the METTL3 promoter and increasing the affinity of HIF1A binding to its promoter, PM2.5 aberrantly upregulated METTL3 expression and increased the m^6^A modification of Baculoviral IAP Repeat Containing 5 (BIRC5). The upregulaion of BIRC5 expression recognized and enhanced by IGF2BP3 could then promote the tumour’s progression, metastasis, and VEGFA-regulated angiogenesis.

Methylation modifications of m^6^A and miRNAs are crucial in human tumours. Some new studies have identified an association between m^6^A modification and miRNA in various tumours (Yi et al., 2020). However, little is known about their association with BC. Yan et al. (Yan et al., 2022) reported the underlying antitumour mechanism of melittin in BC. They demonstrated that the m^6^A modification of miR-146a-5p was responsible for the maturation of pri-miR-146. Through the selective reduction of METTL3 in BC cells, melittin inhibits the miR-146a-5p maturation pathway, suppresses the NOTCH2 pathway, and induces BC cell apoptosis. Han et al. (Han et al., 2019a) confirmed that METTL3 could affect the binding of the microprocessor protein DGCR8 to m^6^A methylated miRNAs, which in turn enhanced DGCR8 recognition and binding to pri-miRNA221/222 in BC, thereby increasing pri-miRNA processing and maturation. Further functional studies showed that miR221/222 directly targeted and inhibited phosphate and tension homology (Pten) expression and promoted tumour progression, and METTL3 plays a cancerous role in BC by positively regulating pri-miR221/222 processes in an m^6^A-dependent way.

It is widely accepted that angiogenesis and tumour cell adhesion are essential in tumour metastasis (Carmeliet and RakeshJain, 2000; Hamidi and Ivaska, 2018). Wang et al. (Wang et al., 2021) revealed that the knockdown of METTL3 suppresses tyrosine kinase endothelial (TEK) and vascular endothelial growth factor A (VEGF-A) expression in BC. The METTL3-mediated modification of m^6^A is necessary to activate TEK/VEGF-A mediated tumour development and angiogenesis in BC. Jin et al. (Jin et al., 2019b) found that the adhesive molecule integrin alpha-6 (ITGA6) was a downstream target of METTL3. The methylated ITGA6 mRNA induced by METTL3 could be recognized by YTHDF1 and YTHDF3, which finally enhanced ITGA6 expression, BC adhesion ability, and malignant phenotypes in BC.

Xie et al. (Xie et al., 2020) demonstrated that SET domain containing 7 (SETD7) and Kruppel-like factor 4 (KLF4), two tumour suppressors, are downstream targets of METTL3 in BC. Further investigations revealed that YTHDF2 could recognize and degrade SETD7 mRNA and KLF4 mRNA methylation by METTL3, which consequently induces BCa progression.

As an oncogene, the CUB domain-containing protein 1 (CDCP1) has emerged as a potential biomarker and therapeutic target in various cancers (Khan et al., 2021). However, the exact mechanism underlying carcinogenesis in BC remains unclear. Yang et al. (Yang et al., 2019) reported that METTL3 and YTHDF1 increased the expression of CDCP1 in BC through a mechanism dependent on the METTL3/CDCP1/YTHDF1 axis, which plays a crucial role in BC tumourigenesis. Ying et al. (Ying et al., 2020) constructed the Cas9-M3 system and achieved targeted mRNA modification. They promote CDCP1 mRNA translation by targeting m^6^A installation onto the 3′untranslated region of CDCP1, facilitating BC development. This approach provides a novel method for targeted interventions for BC and diseases associated with RNA modification defects. Gao et al. (Gao et al., 2020) demonstrated that AF4/FMR2 family member 4 (AFF4) is a direct target of METTL3 and is regulated by METTL3 in an m^6^A-dependent manner. METTL3 enhances the AFF4 RNA’s m^6^A modification and expression in bladder cancer stem cells (BCSCs), which directly controls the expression of MYC and SRY-Box Transcription Factor 2 (SOX2) in BC cells to enhance tumourigenesis in BC. This suggests that AFF4 could be used as a biomarker and therapeutic target for BC patients.

As a key component of m^6^A RNA deposits, METTL14 is identified downregulated in BC and influences tumour aggressiveness. There are currently two opposing views regarding the role of METTL14 in BC. Gu et al. (Gu et al., 2019) found that the knockdown of METTL14 considerably promoted cell proliferation, metastasis, and tumour initiation in bladder tumour-initiating cells (TICs). A positive correlation was observed between METTL14 expression and BC outcomes. However, Catarina et al. (Guimaraes-Teixeira et al., 2022) found that the tumour size and vessel density were considerably reduced by METTL14 protein downregulation in BC cells. They suggested that METTL14 knockdown could impair the tumour aggressiveness because METTL14 could maintain the methyltransferase complex and m^6^A abundance.

TICs are a subpopulation of cancer cells that are indispensable for tumours. Gu et al. (Gu et al., 2019) reported a new regulatory axis in bladder TICs. As a METTL14 downstream target, Notch1 is vital in tumour oncogenesis and TICs self-renewal. The METTL14’s m^6^A modification of Notch1 could decrease the stability of Notch1 mRNA, resulting in the suppression of TICs in BC. The Mettl14-Notch1 pathway may be a potential site for eliminating bladder TICs. Zhang et al. (Zhang et al., 2021a) found that Isorhapontigenin (ISO) could upregulate the expression of METTL14 by activating the transcription factor FOXO3a, and then downregulating the epithelial-to-mesenchymal transition (EMT) marker vimentin in an m^6^A-dependant manner, which in turn inhibits EMT and invasion in BC.

Additionally, WTAP, an m^6^A writer, has been identified as a novel biomarker for the onset and progression of BC, thereby assisting in the diagnosis and prognosis of BC (Chen and Wang, 2018). The expression level of WTAP was significantly overexpressed in BC and associated with poor prognosis (Wei et al., 2021; Wang et al., 2023).

As a novel form of cell death, ferroptosis influences the formation of many tumours, including BC (Chen et al., 2021b; Liu et al., 2021). However, little is known about the association between ferroptosis and m^6^A modification in BC. The endogenous antioxidant factor nuclear factor erythroid 2-related factor 2 (NRF2) is a critical component of tumour ferroptosis. Wang et al. (Wang et al., 2023) identified NRF2 as a remarkable m^6^A site and a downstream target of WTAP. WTAP recognized and enhanced the stability of NRF2 mRNA in a WTAP-YTHDF1-m^6^A-dependent manner, which in turn suppressed the erastin-induced ferroptosis and promoted BC malignancy.

### 4.2 m^6^A erasers and BC

In BC, studies on the m^6^A erasers have mainly focused on the ALKBH5 and FTO. Yu et al. (Yu et al., 2021) demonstrated a reduced expression level of ALKBH5 in BC, which was strongly related to the poor outcomes in BC patients.

As a kind of m^6^A demethylase, the role of FTO in various tumours has been confirmed recently, such as glioblastoma, leukemia, and ovarian cancer (Li et al., 2017). FTO plays different roles in different tumour cell types. It can play an oncogenic role in glioblastoma (Cui et al., 2017); however, it acts as a tumour suppressor gene in ovarian cancer (Huang et al., 2020c). There is also controversy regarding its role in BC. Some studies have demonstrated that FTO is downregulated in BC cell lines than normal bladder cell lines. The upregulated expression of FTO can significantly decrease cell proliferation and invasion; therefore, FTO can exhibit a tumour-suppressing effect on BC (Wen et al., 2020; Yi et al., 2021). In contrast, others reported that FTO was overexpressed in BC, and the knockdown of FTO inhibited bladder cancer cell proliferation (Song et al., 2021; Tao et al., 2021; Zhou et al., 2021; Sun et al., 2022).

Recent studies have depicted that FTO can also regulate the progression of BC cells by affecting the metabolism of miRNAs and lncRNAs. A previous section elucidated that Lnc-MALAT1 could promote progression in the PCa via an m^6^A-dependent mechanism (Mao et al., 2022). According to Tao et al. (Tao et al., 2021), FTO can facilitate suppression of m^6^A modification of the Lnc-Metastasis-associated lung adenocarcinoma transcript 1 (MALAT1) 5′-end and thus prevents YTHDF2’s degradation of MALAT1 mRNA. Thereby enhancing MALAT1 mRNA half-life and expression. This study revealed that FTO promotes the viability and tumourigenesis of BC cells through the FTO/Lnc-MALAT1/miR-384/Mal,T cell differentiation protein 2 (MAL2) axis. Zhou et al. (Zhou et al., 2021) found that FTO regulates miR-576 processing via m^6^A-dependent mechanisms. FTO plays an oncogenic role by regulating the FTO/miR-576/CDK6 pathway in BC by inhibiting miRNA synthesis. They demonstrated that FTO is a potential diagnostic biomarker for BC. Conversely, miRNAs can also affect the FTO process during m^6^A modifications. Sun et al. (Sun et al., 2022) reported that miR-5581–3p is a tumour suppressor in BC, and FTO is regarded as a direct downstream target of miR-5581–3p. miR-5581–3p suppressed FTO synthesis and inhibited BC progression and proliferation via the miR-5581–3p/FTO/Matrix metallopeptidase 9 (MMP9) axis.

Ubiquitin-specific proteases (USPs) are the main members of the deubiquitinase family. Numerous studies have demonstrated that USPs affect tumour progression by regulating the proliferation and death of cancer cells (Pal et al., 2014; Young et al., 2019). Song et al. (Song et al., 2021), for the first time, reported that the post-translational deubiquitination of USP18 could upregulate FTO protein expression and stability. In contrast, FTO can facilitate BC tumourigenesis via its demethylase activity and stabilize the PYCR1 transcripts. Therefore, the USP18/FTO/PYCR1 signaling network may be a potential therapeutic target for BC.

### 4.3 m^6^A readers and BC

In BC, studies on the m^6^A readers have mainly focused on YTHDF1, YTHDF2, YTHDF3, IGF2BP1, and IGF2BP3. Several databases have indicated that YTHDF1 and YTHDF2 mRNA levels were significantly higher in BC tissues than in normal tissues and correlated with poor outcomes (Hu et al., 2021; Zheng et al., 2021). In addition, there was no significant difference in the expression levels of YTHDC1 between tumour and normal tissues; nevertheless, high expression levels of YTHDC1 were associated with a favorable prognosis in BC patients (Chen et al., 2021c). Some studies on YTHDF1-3 in BC have been described in the previous sections (Jin et al., 2019b; Yang et al., 2019; Xie et al., 2020).

According to research, IGF2BPs have a close relationship with BC. IGF2BP1 mRNA levels were significantly higher in BC tissues than in nearby noncancerous tissues and were positively correlated with tumour size and advanced clinical stages of BC (Xie et al., 2021a). By analyzing the TCGA database, Ding et al. (Ding et al., 2022c) demonstrated that IGF2BP2-AS1 expression was elevated in BC tissues and was significantly associated with immune-related factors, indicating that IGF2BP2-AS1 is a potential therapeutic target for immune-related conditions. While other studies have proposed that the expression of IGF2BP2 and IGF2BP3 is significantly higher in BC tissues than in normal bladder tissues and is related to poor prognoses (Huang et al., 2020d; Wu et al., 2021). Xie et al. (Xie et al., 2021a) found that IGF2BP1 was blocked from recognizing downstream m^6^A-modified RNA by circ-PTPRA. By targeting the IGF2BP1/MYC and IGF2BP1/FSCN1 axis, circ-PTPRA inhibits BC progression. As a potential endogenous blocker, circ-PTPRA is expected to broaden the choice for the curative management of BC. Huang et al. (Huang et al., 2020d) reported that IGF2BP3 has an oncogenic effect on human BC progression. By regulating JAK/STAT signaling, IGF2BP3 increases cell proliferation and the cell cycle and inhibits apoptosis in BC cells. There is potential for gene therapy to improve bladder cancer prognosis by targeting IGF2BP3.

## 5 Role of m^6^A regulators in the progression of RCC

**FIGURE 4:**
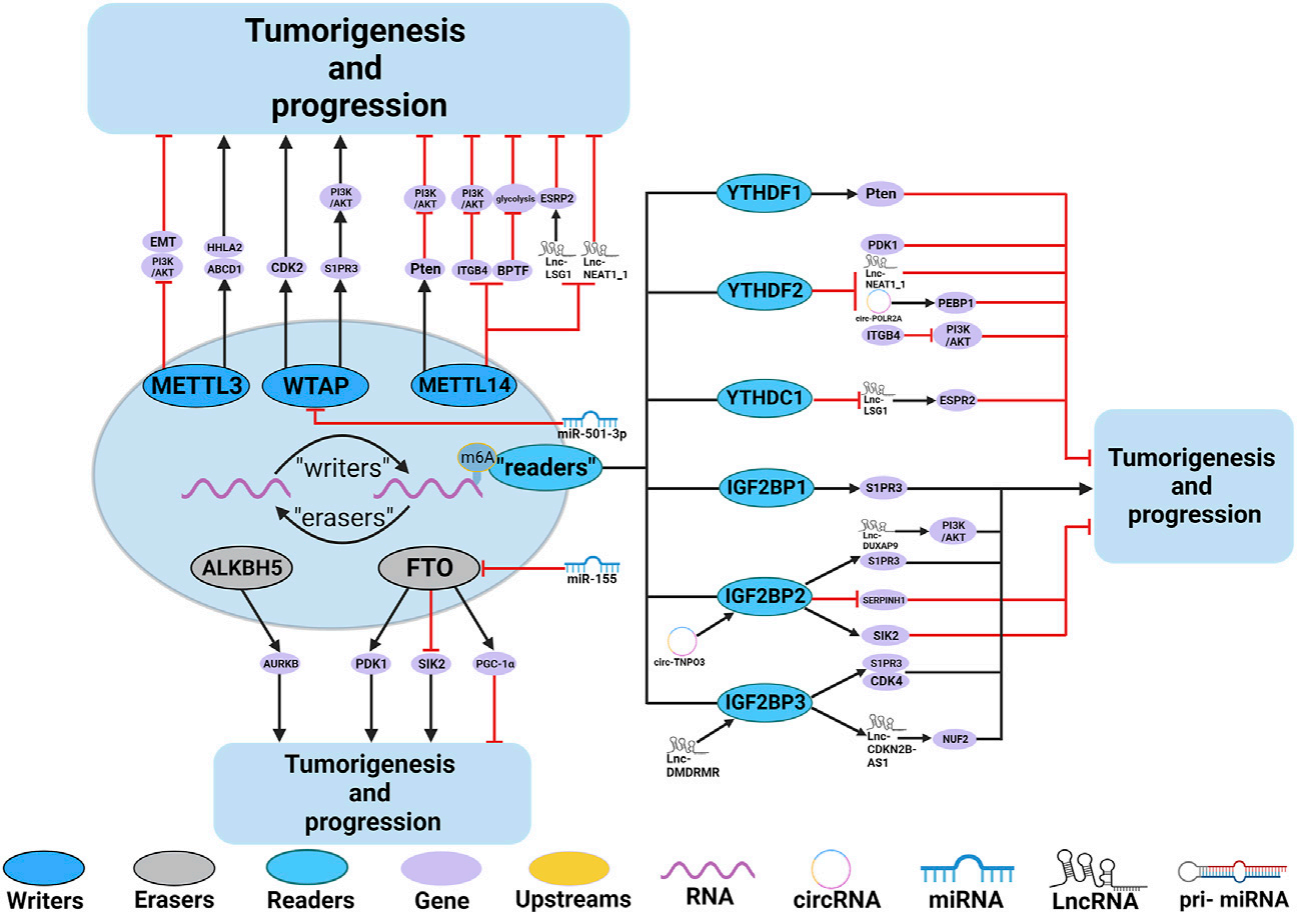
In RCC, m^6^A regulatory proteins contribute to tumorigenesis and progression by interacting with various RNAs. METTL3, METTL14 and WTAP stimulate the progression of RCC by promoting the expression levels of HHLA2, ABCD1, S1PR3 and CDK2. miR-501–3p inhibit the expression of WATP, leading to RCC suppression. METTL14 suppresses the progression of RCC by promoting the expression levels of Pten. METTL3 and METTL14 suppress the progression of RCC by inhibiting the expression levels of EMT, PI3K, BPTF, ITGB4, Lnc-LSG1 and Lnc-NEAT1_1. FTO and ALKBH5 stimulate/inhibit the progression of RCC by affecting the expression levels of AURKB, PDK1, SIK2, and PGC-1α. In addition,miR-155 inhibits the expression of FTO, leading to RCC suppression. IGF2BP1, IGF2BP2 and IGF2BP3 promote the tumorigenesis and progression of RCC by regulating the expression levels of S1PR3, CDK4, Lnc-DUXAP9 and Lnc-CDKN2B-AS1. Lnc-DMDRMR bounds the IGF2BP3 expression and helps stabilize the cell-cycle kinase CDK4, thereby supporting the malignancy state of RCC. YTHDF1, YTHDF2, YTHDC1 and IGF2BP2 inhibit the tumorigenesis and progression of RCC by regulating the expression levels of Pten, ITGB4, PDK1, Lnc-NEAT1_1, Lnc-LSG1, circ-POLR2A, SERPINH1 and SIK2. In addition, circ-TNPO3 interacts with IGF2BP2 to destabilize the mRNA of SERPINH1, thereby inhibiting the tumorigenesis and progression of RCC.

Numerous studies have demonstrated the participation of m^6^A regulators in various functions in the RCC, such as dysregulation of signaling pathways and regulation of autophagy. The roles and mechanisms of m^6^A regulators in RCC are summarized in Figure 4 and Table 3.

**TABLE 3:** The role and mechanism of m6A regulators in RCC.

m6A Regulators	Roles in m6A	Expression in cancer	Mechanism	Function in cancer	References
METTL3	Writer	upregulation	METTL3/HHLA2	Promotes tumorigenesis and progression	Shen et al. (2022a)
METTL3	Writer	upregulation	METTL3/ABCD1	Promotes tumorigenesis and progression	Liu et al. (2022d)
METTL3	Writer	downregulation	METTL3/EMT	Inhibits tumorigenesis and development	Zhang et al. (2021b)
METTL3	Writer	downregulation	METTL3/PI3K/Akt/mTOR	Inhibits tumorigenesis and development	Zhang et al. (2021b)
METTL14	Writer	downregulation	METTL14/Lnc-LSG1/ESRP2/YTHDC1	Inhibits tumour invasion and migration	Zhang et al. (2022)
METTL14	Writer	downregulation	METTL14/Lnc-NEAT1_1/YTHDF2	Inhibits tumour progression and migration	Liu et al. (2022d)
METTL14	Writer	downregulation	METTL14/BPTF/glycolysis	Inhibits tumour invasion and migration	Chen et al. (2022)
METTL14	Writer	downregulation	METTL14/Pten/PI3K/AKT/YTHDF1	Inhibits tumour proliferation and migration	Laurette et al. (2020)
METTL14	Writer	downregulation	METTL14/ITGB4/PI3K/AKT/YTHDF2	Inhibits tumour progression and migration	Koedoot et al. (2019)
WTAP	Writer	upregulation	WTAP/S1PR3/PI3K/AKT/IGF2BPs	Promotes tumo tumorigenesis and migration	Xu et al. (2022a)
WTAP	Writer	upregulation	miR-501–3p/WTAP/CDK2	Promotes tumour progression	Shen et al. (2022b)
ALKBH5	Eeaser	upregulation	ALKBH5/AURKB	Promotes tumour progression	Yang et al. (2022)
FTO	Eraser	upregulation	FTO/autophagy/SIK2/IGF2BP2	Promotes tumour tumorigenesis and curbs autophagy	Levine (2007)
FTO	Eraser	upregulation	FTO/PDK1/YTHDF2	Promotes tumour progression and migration	von Hagen et al. (2021)
FTO	Eraser	downregulation	FTO/PGC‐1α	Inhibits tumour proliferation	Xu et al. (2022b)
FTO	Eraser	downregulation	miR-155/FTO	Inhibits tumour proliferation and promotes tumour apoptosis	Li et al. (2022b)
YTHDF1	Reader	-	METTL14/Pten/PI3K/AKT/YTHDF1	Inhibits tumour proliferation and migration	Laurette et al. (2020)
YTHDF2	Reader	downregulation	METTL14/Lnc-NEAT1_1/YTHDF2	Inhibits tumour progression and migration	Liu et al. (2022d)
YTHDF2	Reader	downregulation	METTL14/ITGB4/PI3K/AKT/YTHDF2	Inhibits tumour progression and migration	Koedoot et al. (2019)
YTHDF2	Reader	downregulation	FTO/PDK1/YTHDF2	Inhibits tumour progression and migration	von Hagen et al. (2021)
YTHDF2	Reader	downregulation	YTHDF2/circ-POLR2A/PEBP1	Inhibits tumour progression and migration	Tan et al. (2021)
YTHDC1	Reader	-	METTL14/Lnc-LSG1/ESRP2/YTHDC1	Inhibits tumour invasion and migration	Zhang et al. (2022)
IGF2BPs	Reader	upregulation	WTAP/S1PR3/PI3K/AKT/IGF2BPs	Promotes tumour tumorigenesis and migration	Xu et al. (2022a)
IGF2BP2	Reader	-	IGF2BP2/Lnc-DUXAP9/PI3K/Akt	Promotes tumour proliferation and motility	Basu et al. (2021)
IGF2BP2	Reader	downregulation	circ-TNPO3/IGF2BP2/SERPINH1	Inhibits tumour progression and migration	Zhong et al. (2023)
IGF2BP2	Reader	-	FTO/autophagy/SIK2/IGF2BP2	Inhibits tumour tumorigenesis and promotes autophagy	Levine (2007)
IGF2BP3	Reader	upregulation	IGF2BP3/Lnc-CDKN2B-AS1/NUF2	Promotes tumour tumorigenesis and progression	Jiang et al. (2022)
IGF2BP3	Reader	upregulation	Lnc-DMDRMR/IGF2BP3/CDK4	Promotes tumour tumorigenesis and progression	Lenis et al. (2020)

### 5.1 m^6^A writers and RCC

Studies on RCC have mostly focused on the regulatory factors METTL3 and METTL14. Numerous studies have indicated that METTL3 has two roles in RCC. Most studies found that METTL3, an oncogene, was upregulated in RCC compared to normal tissues and correlated with poor outcomes (Zhang et al., 2020a; Chen et al., 2020b; Wang et al., 2020c; Zhao et al., 2020). An oncogene, the human endogenous retrovirus-H long terminal repeat-associating protein 2 (HHLA2), is an important prognostic predictor in RCC (Chen et al., 2019b). According to Zhu et al. (Zhu et al., 2022), through m^6^A modification of HHLA2 mRNA, METTL3 regulates and promotes HHLA2 expression, thereby enabling RCC growth and invasion. Shi et al. (Shi et al., 2021) reported that ABCD1 (an ATP-binding cassette transporter of fatty acids) is a key downstream target of METTL3 in RCC, and the knockdown of ABCD1 in RCC cells decreased cancer cell migration and spheroid formation. METTL3 enhances ABCD1 translation in an m^6^A-dependent manner to promote RCC.

Although most studies provide evidence that METTL3 contributes to the proliferation and progression of RCC, reports have shown a tumour suppressor role of METTL3. Li et al. (Xiao et al., 2017) indicated that METLL3 acts as a tumour suppressor, and the knockdown of METTL3 considerably promotes RCC proliferation. According to their findings, EMT pathways may contribute to the underlying mechanism of RCC and upregulated METTL3 may reverse EMT progression. In addition, they demonstrated that through its negative impact on PI3K/Akt/mTOR signaling, METTL3 might influence RCC progression.

Like BC, the precise role of METTL14 in RCC remains controversial. Most studies have demonstrated that lower METTL14 expression in RCC tissues is negatively associated with a poor prognosis. METTL14 can act as a tumour suppressor (Zhang et al., 2021b; Shen et al., 2022a; Liu et al., 2022c; Liu et al., 2022d; Zhang et al., 2022). However, some researchers have opposite conclusions (Chen et al., 2022).

Methylation modifications of lncRNAs and m^6^A play an important role in tumours. Studies have demonstrated that METTL14 can also regulate the growth of RCC cells by influencing the metabolism of lncRNAs. According to a new study in 2022 (Shen et al., 2022a), the Lnc-LSG1 was identified as a downstream target of METTL14, and it could promote RCC progression and metastasis by binding to ESRP2 and promoting ESRP2 ubiquitination and degradation. METTL14 inhibited the interaction between epithelial splicing regulatory protein 2 (ESRP2) and Lnc-LSG1 by increasing the binding of YTHDC1 to the m^6^A site in Lnc-LSG1. The overexpression of METTL14 considerably decreases the migration and invasion abilities of RCC cells. Similarly, Liu et al. (Liu et al., 2022c) found that RCC tissues showed higher levels of Lnc-NEAT1_1, which was negatively correlated with METTL14 expression. Further studies have demonstrated that METTL14 suppresses the expression of NEAT1_1 in an m^6^A-dependent manner via YTHDF2-dependent RNA degradation, thereby inhibiting the progression and metastasis of RCC. These studies presented the first insight into how m^6^A-modified lncRNAs function and may set the stage for novel biomarkers and approaches in RCC.

The bromodomain PHD finger transcription factor (BPTF) plays a significant role in the invasion and progression of cancers (Koedoot et al., 2019; Laurette et al., 2020). Nevertheless, very few studies have investigated the transcriptional or post-transcriptional regulation of BPTF, particularly m^6^A modification. In a groundbreaking study, Zhang et al. (Zhang et al., 2021b) found a negative correlation between the stability of BPTF mRNA and the modification of m^6^A in the BPTF 3′UTR regions by METTL14 in RCC. The deficiency of METTL14 increased the expression of BPTF, which accelerated RCC metastasis. A subsequent study has demonstrated that the METTL14/BPTF axis contributes to RCC metastasis by increasing glycolysis. They indicated that BPTF inhibitor AU1 may be a promising therapeutic candidate for overexpressing BPTF in RCC.

Many signaling pathways associated with tumour development have been recognized as m^6^A targets and are controlled by m^6^A modifiers. The PI3K/AKT pathway plays an essential role in various cellular processes, including the growth and survival of RCC cells (Sato et al., 2013; Zhu et al., 2020). Recently, the mechanism of METTL14 and WTAP methylation effects on tumour proliferation through the PI3K-AKT pathways in RCC has been demonstrated. According to Zhang et al. (Zhang et al., 2022), Pten is a downstream target of METTL14 in RCC. By regulating Pten mRNA for m^6^A modification via a YTHDF1-dependent mechanism, the downregulation of METTL14 in RCC inhibited Pten expression, leading to the progression of tumours through PI3K/AKT signaling. The Integrinβ4 (ITGB4) is a transmembrane receptor involved in the tumourigenesis and the invasiveness of several malignancies (Lipscomb et al., 2005; Ruan et al., 2020). Nonetheless, the underlying mechanisms in RCC remain unclear. Liu et al. (Liu et al., 2022d) reported that significant overexpression of ITGB4 in RCC tissues and metastases, together with poor prognosis, is associated with high levels of ITGB4. Further investigations revealed that a METTL14-mediated modification of m^6^A negatively regulates ITGB4 expression. During this process, YTHDF2 preferentially interacts with the ITGB4 mRNA and promotes its degradation. Downregulation of METTL14 and overexpression of ITGB4 promote tumour invasion, metastasis, and the PI3K/AKT signaling in RCC.

WTAP is a crucial gene involved in many tumour diseases. A relationship between WTAP and RCC was also identified. According to recent studies, WTAP is significantly overexpressed in RCC cell lines and tissues, and higher WTAP levels lead to a poor prognosis in patients with RCC (Tang et al., 2018; He et al., 2021; Ying et al., 2021). Ying et al. (Ying et al., 2021) reported that WTAP and IGF2BP proteins enhanced S1PR3 stability in an m^6^A-dependent manner and regulated its PI3K/AKT pathway, which promoted tumourigenesis and migration. He et al. Reported that the interaction of miRNA with the WTAP affects RCC proliferation and development (He et al., 2021). Studies have indicated that miR-501–3p is a latent suppressor that is downregulated in RCC. Further investigation revealed that WTAP is a direct downstream target gene of miR-501–3p and is inhibited by it. The overexpressed WTAP may play an oncogenic role through the miR-501–3p/WTAP/Cyclin Dependent Kinase 2 (CDK2) axis in RCC.

### 5.2 m^6^A eraser and RCC

Studies of m^6^A erasers in the RCC have focused on ALKBH5 and FTO. Recent studies have provided insight into the regulation of ALKBH5 methylation in RCC. They concluded that ALKBH5 had higher expression levels in both RCC tumour tissues and cell lines. (Chen et al., 2020b; Guimaraes-Teixeira et al., 2021). Zhang et al. (Zhang et al., 2020b). reported that ALKBH5 could play an oncogenic role in the tumourigenesis of RCC, and that Aurora Kinase B (AURKB) expression was positively correlated with the expression of ALKBH5 in RCC tissues. By demethylating the mRNA of AURKB mRNA, ALKBH5 enhances the AURKB stability and promotes RCC cell proliferation in an m^6^A-dependent manner.

Depending on the context, FTO is believed to play both tumour-suppressing and cancer-promoting roles in an m^6^A-dependent manner in solid tumours. Among the four studies discussed in detail in the subsequent sections, two identified FTO as carcinogenic (Xu et al., 2022a; Shen et al., 2022b), whereas the other two suggested that it is a carcinogenic tumour suppressor (Zhuang et al., 2019; Yang et al., 2022).

Autophagy has been implicated in various pathophysiological processes, including tumourigenesis, proliferation, and cell death (Levine, 2007). Excitingly, Xu et al. reported that the m^6^A modification participates in autophagy (Xu et al., 2022a). Salt-inducible kinase 2 (SIK2) is identified as a functional target of m^6^A-mediated autophagy in RCC. FTO demethylates SIK2, inhibits the stability of SIK2 through an m^6^A-IGF2BP2-dependent mechanism, and plays a crucial role in inhibiting autophagy and promoting carcinogenicity in RCC. High levels of FTO were also found in the tumour tissues and databases of RCC patients. (Shen et al., 2022b). By protecting Pyruvate Dehydrogenase Kinase 1 (PDK1) mRNA from degradation caused by YTHDF2, FTO promotes the progression and migration of RCC in an m^6^A-dependent manner. RCC may benefit from the therapeutic targeting of the FTO/YTHDF2/PDK1/m^6^A axis.

In another study, FTO was found to be downregulated in Von Hippel-Lindau (VHL)-deficient RCC tissue and correlated with tumour severity and poor prognosis (Zhuang et al., 2019). Further investigation confirmed that PPARg coactivators-1α (PGC-1α) was a functionally important target of FTO. Overexpression of FTO in VHL-deficient RCC restored mitochondrial activity, induced oxidative stress and ROS production, and restrained tumour proliferation by increasing the expression of PGC-1α. Therefore, identifying a novel FTO/PGC-1 axis represents a potential therapeutic target for VHL-deficient RCC.

Interactions between m^6^A modification of FTO and miRNAs in RCC have been demonstrated. Zhuang et al. (Yang et al., 2022) observed that miR-155 expression was considerably higher in RCC tissues than in adjacent normal tissues. Further studies confirmed that miR-155 inhibited FTO expression and increased the global m^6^A levels while promoting tumour cell progression and decreasing apoptosis in an FTO-dependent manner.

### 5.3 m^6^A reader and RCC

Studies of m^6^A readers in RCC have mainly focused on the YTHDF1, YTHDF2, YTHDC1, and IGF2BPs. Several aspects of the role and mechanism of YTHDF1, YTHDF2, YTHDC1, and IGF2BPs in RCC have been explained in the previous sections (Ying et al., 2021; Shen et al., 2022a; Xu et al., 2022a; Shen et al., 2022b; Chen et al., 2022; Liu et al., 2022c; Liu et al., 2022d; Zhang et al., 2022). Recent studies have reported that YTHDF1-3 could act as tumour markers in RCC. Felix et al. (von Hagen et al., 2021) found that YTHDF1-3 were significantly downregulated in RCC compared to normal tissue and were correlated with patients’ clinical M stage. Xu et al. (Xu et al., 2022b) reported that circ-POLR2A serves as an oncogene by enhancing the ubiquitination and degradation of Phosphatidylethanolamine Binding Protein 1 (PEBP1). YTHDF2 played a suppressive part in the expression of circ-POLR2A in an m^6^A-dependent manner.

YTHDC1 was downregulated in RCC tissues compared to that in normal tissues and had a predictive value for the risk of RCC. Low levels of YTHDC1 been linked to poor outcomes. This highlights the diagnostic and prognostic value of YTHDC1 in RCC (von Hagen et al., 2021; Li et al., 2022b).

IGF2BPs are principal members of the reader protein of the N6-Methyladenosine RNA Methylation family; however, their function and content in RCC remain controversial. Studies suggest that IGF2BP1-3 were overexpressed in RCC compared to adjacent normal controls and were associated with poor patient prognoses (Zhang et al., 2021c; Ying et al., 2021; Xing et al., 2022). Recent studies have also identified the interactions of lncRNAs and miRNAs with the IGF2BPs in RCC. Tan et al. (Tan et al., 2021) reported that Lnc-Double Homeobox A Pseudogene 9 (DUXAP9) is significantly upregulated in localized RCC and is closely associated with poor prognosis. IGF2BP2 binds to DUXAP9 through its m^6^A modification sites, which act as m^6^A readers and promote the stability of DUXAP9. They concluded that Lnc-DUXAP9 might promote tumour proliferation via IGF2BP2/Lnc-DUXAP9/PI3K/Akt axis. Pan et al. (Pan et al., 2022) reported that circ-ransportin-3 (TNPO3) was significantly downregulated in RCC using TCGA database and *in vivo* experiments. Further tests proved that cicr-TNPO3 could downregulate Serpin family H member 1 (SERPINH1) expression by interacting with IGF2BP2 through m^6^A modification, thereby inhibiting RCC metastasis. Xie et al. (Xie et al., 2021b) showed that IGF2BP3 promotes Ndc80 kinetochore complex component (NUF2) expression and RCC progression by enhancing the stability of cyclin-dependent kinase inhibitor 2 B antisense 1 lncRNA (CDKN2B-AS1). Gu et al. (Gu et al., 2021) reported that a high co-expression level of DMDRMR (a novel DNA methylation-deregulated and RNA m^6^A reader-cooperating lncRNA) and IGF2BP3 is associated with poor prognosis in RCC patients. IGF2BP3 which bind Lnc-DMDRMR to stabilize the cell-cycle kinase CDK4 in an m^6^A-dependent manner, ultimately enhancing the G1-S transition and promoting tumour progression in RCC.

## 6 Role of m^6^A regulators in urologic tumours drug resistance

Increasing studies have shown a significant correlation between m^6^A modification and drug resistance in urologic tumours, suggesting the potential of targeting m^6^A in urologic tumours with therapeutic resistance. The regulation for m^6^A levels in urologic tumours maybe a cut-point for chemotherapy, targeted therapy and other drug therapy. We summarizes current studies showing that m^6^A regulators are closely related to drug resistance and aims to provide deeper insights into tumourigenesis or the drug resistance mechanism of urologic tumours. The role of m6A modification in urologic tumours drug resistance are summarized in Table 4.

**TABLE 4:** The role of m6A modification in urologic tumours drug resistance.

Cancer types	m6A Regulators	Roles in m6A	Mechanism	Function in cancer	References
PCa	METTL3	Writer	METTL3/NR5A2	ARSI resistance	Cotter et al. (2021)
PCa	METTL3	Writer	METTL3/circ-RBM33/FMR1/PDHA1	ARSI resistance	Zhong et al. (2023)
PCa	YTHDC1	Reader	SLC12A5/YTHDC1/HOXB13	ARSI resistance	Yuan et al. (2023)
PCa	IGF2BP2	Reader	IGF2BP2/circ-ARHGAP29/LDHA	docetaxel resistance	Jiang et al. (2022)
BC	WTAP	Writer	circ-0008399/WTAP/TNFAIP3	cisplatin resistance	Wei et al. (2021)
BC	VIRMA	Writer	VIRMA/circ-MORC3/global m6A levels	cisplatin resistance	Su and Tianxin (2020)
BC	ALKBH5	Eraser	ALKBH5/CK2 α	cisplatin resistance	Yu et al. (2021)
BC	YTHDC1	Reader	YTHDC1/PTEN/PI3K/AKT	cisplatin resistance	Su et al. (2023)
RCC	METTL14	Writer	METTL14/TRAF1/IGF2BP2	sunitinib resistance	Chen et al. (2022)
RCC	YTHDC1	Reader	HDAC2/YY1/YTHDC1/ANXA1	sunitinib resistance	Li et al. (2022b)

### 6.1 m^6^A modification and drug resistance in PCa

Androgen deprivation therapy (ADT) is the mainstream therapy strategy for PCa patients. Although initially effective, most of patients progress to castration-resistant prostate cancer (CRPC) after a few years of treatment. CRPC finally develops resistance to second generation of anti-androgen agents. Therefore, identification of the mechanism underlying androgen receptor signaling inhibitors (ARSI) may provide new effective treatments for PCa. Yuan et al. (Yuan et al., 2023) reported that SLC12A5 (a neuron-specific potassium-chloride co-transporter) is significantly upregulated in CRPC compared to prostate adenocarcinoma samples and normal counterparts and promotes castration resistance. Further mechanism studies elucidated that SLC12A5 bind with m^6^A reader YTHDC1 and in turn promotes the expression of transcription factor HOXB13 to promote tumour progression and castration resistance in an m^6^A-dependent manner. Cotter et al. (Cotter et al., 2021) found that METTL3 is downregulated in advanced metastatic CRPC compared with benign prostate tissue. They then demonstrated a negative correlation between METTL3 and NR5A2 (a hepatocyte nuclear factor) in CRPC patients. It has been speculated that the upregulation of NR5A2 promote CRPC cells resistant to androgen receptor antagonists via an androgen receptor–independent mechanism. This result provides a new mechanism for m^6^A in regulating drug resistance of ARSI.

Unlike normal cells, cancer cells are more dependent on glycolysis even under aerobic conditions. However, a latest study indicated that oxidative phosphorylation is substantially increased in CPRC cells compared to androgen sensitive PCa cells (Basu et al., 2021). According to Zhong et al. (Zhong et al., 2023), circ-RBM33 was upregulated and played a oncogenic role in PCa. The knockdown of circ-RBM33 increased the response sensitivity of PCa cells to ARSI treatment. Mechanistically, METTL3 methylated circ-RBM33 and promoted the interaction between circ-RBM33 and FMR1 in an m^6^A manner, which enhanced mitochondrial respiration by regulating the expression of Pyruvate dehydrogenase alpha 1 (PDHA1). Therefore, METTL3 and circ-RBM33 may function as potential targets for future CPRC therapy.

Docetaxel (DTX) is the first-line chemotherapy for metastatic prostate cancer. However, its therapeutic effect could be limited due to acquired drug resistance in PCa and recurrence may appear after several years of treatment. Jiang et al. (Jiang et al., 2022) showed that circ-ARHGAP29 expression was markedly elevated in PCa docetaxel-resistant cells compared to docetaxel-sensitive cells. The circARHGAP29 could promote the expression of Lactate dehydrogenase A (LDHA) and glycolysis by interacting with m^6^A reader IGF2BP2, thus increasing docetaxel resistance.

### 6.2 m^6^A modification and drug resistance in BC

Chemotherapy is the first-line treatment for muscle-invasive and metastatic bladder cancer. However, resistance to chemotherapy often results in treatment failure in BC (Lenis et al., 2020). Therefore, the mechanism of resistance to chemotherapy in BC requires further investigation.

A recent study reported that YTHDC1 plays a pivotal role in cisplatin resistance of bladder cancer. Su et al. (Su et al., 2023) revealed that the expression of YTHDC1 in cisplatin-resistant BC tissues is lower compared to the cisplatin-sensitive cancer tissues. In BC, YTHDC1 could promote the PTEN mRNA stability and expression by interacting with it and, in turn, inhibting the PI3K/AKT signalling. Wei et al. (Wei et al., 2021) reported that circ-0008399 could bind to WTAP to promote the constitution of the m^6^A methyltransferase complex and then increase the mRNA stability of TNF alpha-induced protein 3 (TNFAIP3) in an m^6^A-dependent manner. They confirmed that the circ-0008399/WTAP/TNFAIP3 axis might be crucial in developing cisplatin resistance in BC. Su et al. (Su and Tianxin, 2020) found a positive correlation between upregulated circ-MORC3 and cisplatin-resistance in BC tissues. circ-MORC3 could bind to the m^6^A reader VIRMA and increase global m^6^A levels, which then promote cisplatin resistance by enhancing DNA repair. According to a recent study (Yu et al., 2021), ALKBH5 could hinder the glycolytic pathway by reducing the stability of casein kinase 2 α(CK2 α) mRNA in an m^6^A-dependent manner, thus inhibiting the progression of BC and promoting BC cell sensitivity to cisplatin.

### 6.3 m^6^A modification and drug resistance in RCC

The mechanism of tumour resistance involves numerous factors in RCC, including angiogenic switching, gene mutations, and modifications of the tumour microenvironment (Makhov et al., 2018). In RCC, the METTL14 is involved in drug resistance by upregulating the tumour necrosis factor receptor-associated factor 1 (TRAF1) expression (Chen et al., 2022). Chen et al. identified that METTL14 was upregulated and acted as an oncogene in RCC, with a distinct role in other known studies. Moreover, a positive correlation was observed between METTL14 and TRAF1 expression in sunitinib-resistant tissues. Further investigation showed that METTL14-mediated m^6^A modification enhanced the stability of TRAF1 mRNA in an IGF2BP2-dependent manner and positively modulated sunitinib resistance by regulating apoptosis and angiogenesis.

The association between Annexin-A1 (ANXA1) and the chemosensitivity of tyrosine kinase inhibitors has been illustrated by many recent studies (Chuang et al., 2021; Xiong et al., 2021). Interestingly, Li et al. reported that the reader YTHDC1 regulates ANXA1 in RCC (Li et al., 2022b). ANXA1 is recognized as a downstream target of YTHDC1 in RCC. By inhibiting ANXA1, YTHDC1 suppressed RCC MAPK signaling and inhibited RC cell proliferation. Further studies confirmed that YTHDC1-ANXA1 axis could control sunitinib sensitivity in RCC. The YY1/HDAC2 complex could inhibit the expression of YTHDC1 and promot sunitinib sensitivity via HDAC2/YY1/YTHDC1/ANXA1 axis in RCC. ANXA1 is an important target of m^6^A modification in developing drug resistance to sunitinib therapy in RCC.

## 7 Discussion

The role of m^6^A modification in tumour mechanisms and treatment has become a hot topic in epigenetics. It has been found that m^6^A modification affects every aspect of urologic tumours. This review comprehensively summarized the m^6^A methylation modifications in PCa, BC, and RCC based on tumour mechanisms and developmental aspects. It should be noted that the roles of m^6^A regulation in urologic tumours are complex and even inconsistent. For example, Zhu et al. (Zhu et al., 2022)reported that the expression of METTL3 is upregulated in RCC tissues compared with normal tissues. Poor prognosis was associated with high levels of METTL3 in patients with RCC. By contrast, Li et al. (Xiao et al., 2017)showed that METTL3 inhibits RCC progression through its negative impact on PI3K/Akt/mTOR signaling in an m^6^A-dependent manner. In addition, The same kind of m^6^A regulators can exert different functions in different tumours. These contradicting results can be attributed to tumour heterogeneity, differences in experimental setups and different ethnic. For instance, Lewis et al. (Lewis et al., 2010) reported the rs9939609 in FTO was negatively associated with risk of PCa in white European cohort. Beyond this, the target gene expression modified by m^6^A should depend not only on the “writers” and “erasers” but also on the functions of “readers”. These may explain the contradictory results between “writers” and “erasers” previously reported. In the future, more studies on the regulatory mechanisms of m^6^A are quite needed.

Apparently, notable progress has been achieved in exploring the role of m^6^A modification of urologic tumours. m^6^A regulators have been reported to serve as potential biomarkers for the early diagnosis and prognosis of urologic tumours. For instance, as has been previously noted, METTL3 was significantly upregulated in BC and associated with poor prognosis, it may be a novel promising prognostic biomarker for BC. Among the studies summarized, we supposed that the global m^6^A levels serve as biomarker for diagnosis of urologic tumours are limited and unreliable. Because we surprisingly found that both “writers” (METTL3, METTL14 and WTAP) and “erasers” (ALKBH5 and FTO) could abnormally overexpressed and exert oncogenic functions or downregulated and play tumour suppressor roles in urologic tumours. In comparison, the target genes modified by m^6^A are more appropriate to serve as biomarkers. However, although with the advancement of high-throughput sequencing mass spectrometry technology, increasing target genes have been identified in m^6^A modification of tumours (Dominissini et al., 2012; Meyer et al., 2012; Linder et al., 2015). The m^6^A modification detection technology is still characterized by large amounts of RNA requirement, high cost and low precision. Therefore, continuous refinement of m^6^A detection methods and analytical techniques are warranted. It would be helpful to further explore and clarify the use of m^6^A target transcripts as potential diagnosis biomarkers of urologic tumours. As m^6^A detection techniques and research methods continue to progress, we believe in the future the improved methods of m^6^A modification would make it possible to screening early tumour, thus reducing urologic tumours mortality.

The critical roles of m^6^A modification in urologic tumours also showed that m^6^A regulators have an enormous potential as novel antitumour therapeutic targets. Small molecule inhibitors and activators promise to treat urologic tumours by inhibiting or compensating the expression of abnormal m^6^A regulators. Discussion to date, there have been a few studies that reported the development of inhibitors that target m^6^A regulators in urologic tumours. UZH2 is recently developed and is a potent and selective inhibitor for METTL3. It exhibited interesting antitumours activity in PCa cell lines (Dolbois et al., 2021). Meclofenamic acid is a highly selective FTO inhibitor, it could be combined with nanoplatform based on gold nanorods and promote photothermal immunotherapy in PCa (Liu et al., 2022e). Future studies should work toward to the development of more specific and potent m^6^A regulatiors inhibitors and activators by high throuput screening, structure-based virtual screening and ligand-based virtual screening. In addition, just as the abovementioned, dysregulation of m^6^A regulators also serves a key role in urologic tumours drug resistance, which could provide foundations for treating cancer resistant research in the future.

There are still some issues that need to be resolved since m^6^A modification in urologic tumours has only recently gained attention as a critical epigenetic regulator. For instance, most of the available urologic tumour studies have focused on popular methyltransferases and m^6^A binding proteins, such as METTL3, METTL14, YTHDFs and IGF2BPs. However, only a small amount of research has conveyed the directions for future exploration of “erasers”. Moreover, the role and aberrant expression of some m^6^A regulators in tumours remain controversial. Further investigations are required to fully resolve these issues. Thirdly, most of the research have been molecular mechanisms and basic research of m^6^A regulators, with a lack of clinical studies and applications. Moreover, the probable side effects of m^6^A modification in urologic tumours are unclear and should be clarified with further detailed studies.

## 8 Conclusion

m^6^A modification as a research hotspot in epigenetics have been rapidly developed during recent years. The recent research highlight the important role of m^6^A modification not only in tumorigenesis and progression, but also in regulating drug resistance. However, the specific and detailed molecular mechanisms of urologic tumours remain unclear, related studies to m^6^A modification are still in preliminary stage. Undeniably, m^6^A modification show great potential in the treatment of tumours, which requires more advanced technology and studies. Previous studies on m^6^A modification have paved the way for future research. It is essential to understand the abnormal characteristics of urologic tumours by elucidating the mechanisms of post-transcriptional m^6^A modifications. By clarifying these issues, we will gain a more in-depth understanding of the pathogenic mechanisms of PCa, BC, and RCC and provide more feasible treatment strategies targeting m^6^A modification.

## Figures and Tables

**Table:** 

**PCa**	Prostate cancer
**BC**	Bladder cancer
**RCC**	Renal cell cancer
**m** ^**6**^ **A**	N6-methyladenosine
**METTL3**	Methyltransferase-like 3
**METTL14**	Methyltransferase-like 14
**WTAP**	Wilm’s tumour-associated protein
**METTL16**	Methyltransferase-like 16
**VIRMA**	Vir-like m^6^A methyltransferase-associated protein
**ZC3H13**	Zinc finger CCCH domain-containing protein 13
**RBM15**	RNA binding pattern protein 15
**ALKBH5**	α-ketoglutarate-dependent dioxygenase homolog 5
**FTO**	Fat mass and obesity-related protein
**IGF2BP**	Insulin-like growth factor 2 mRNA binding protein
**YTH**	YT521-B homology
**ncRNAs**	Non-coding RNAs
**PVT1**	Plasmacytoma variant translocation 1
**BLM**	Bloom syndrome protein
**KIF3C**	Kinesin Family Member 3C
**MALAT1**	Metastasis-associated lung adenocarcinoma transcript 1
**SNHG7**	Small nucleolar RNA host gene 7
**SRSF1**	Serine/arginine-rich splicing factor 1
**LHPP**	Phospholysine phosphohistidine inorganic pyrophosphate phosphatase
**NKX3-1**	NK3 Homeobox 1
**USP4**	Ubiquitin Specific Peptidase 4
**ARHGDIA**	Rho GDP dissociation inhibitor α
**LEF1**	Lymphoid Enhancer-binding Factor
**GLI1**	Glioma-associated Oncogene Homolog 1
**TMEs**	Tumour microenvironments
**LXA4**	Lipid molecule lipoxin A4
**STAT6**	Signal transducers and activators of transcription 6
**ITGB1**	Integrin β1
**THBS1**	Thrombospondin 1
**EIF3C**	Eukaryotic Initiation Factor 3
**MC4R**	Melanocortin-4 receptor
**CLIC4**	Chloride intracellular channel 4
**PLK1**	Polo-like kinase 1
**KDM5a**	Lysine demethylase 5a
**MOB3B**	MOB kinase activator 3B
**IGF1R**	Type 1 insulin-like growth factor receptor
**MID1**	Midline-1
**PD-L1**	Programmed death ligand 1
**PM2.5**	Particulate matter
**BIRC5**	Baculoviral IAP Repeat Containing 5
**Pten**	Phosphate and tension homology
**TEK**	Tyrosine kinase endothelial
**VEGF-A**	Vascular endothelial growth factor A
**ITGA6**	Integrin alpha-6
**SETD7**	SET domain containing 7
**KLF4**	Kruppel-like factor 4
**CDCP1**	CUB domain-containing protein 1
**AFF4**	AF4/FMR2 family member 4
**BCSCs**	Bladder cancer stem cells
**SOX2**	SRY-Box Transcription Factor 2
**TICs**	Tumour-initiating cells
**ISO**	Isorhapontigenin
**EMT**	Epithelial-to-mesenchymal transition
**TNFAIP3**	TNF alpha-induced protein 3
**NRF2**	Nuclear factor erythroid 2-related factor 2
**CK2α**	**c**asein kinase 2 α
**MALAT1**	Metastasis-associated lung adenocarcinoma transcript 1
**MAL2**	Mal,T cell differentiation protein 2
**MMP9**	Matrix metallopeptidase 9
**USPs**	Ubiquitin-specific proteases
**HHLA2**	Human endogenous retrovirus-H long terminal repeat-associating protein 2
**ABCD1**	An ATP-binding cassette transporter of fatty acids
**ESRP2**	Epithelial splicing regulatory protein 2
**BPTF**	Bromodomain PHD finger transcription factor
**TRAF1**	Tumour necrosis factor receptor-associated factor 1
**ITGB4**	Integrinβ4
**CDK2**	Cyclin Dependent Kinase 2
**AURKB**	Aurora Kinase B
**SIK2**	Salt-inducible kinase 2
**PDK1**	Pyruvate Dehydrogenase Kinase 1
**PGC-1α**	PPARg coactivators-1α
**PEBP1**	Phosphatidylethanolamine Binding Protein 1
**ANXA1**	Annexin-A1
**DUXAP9**	Double Homeobox A Pseudogene 9
**TNPO3**	Transportin-3
**SERPINH1**	Serpin family H member 1
**NUF2**	Ndc80 kinetochore complex component
**ADT**	Androgen deprivation therapy
**CRPC**	castration-resistant prostate cancer
**ARSI**	androgen receptor signaling inhibitors
**PDHA1**	Pyruvate dehydrogenase alpha 1
**CDKN2B-AS1**	cyclin-dependent kinase inhibitor 2B antisense 1
